# Patient Journey, Treatment Patterns, and Disease Burden of Patients With Idiopathic Hypereosinophilic Syndrome

**DOI:** 10.1002/jha2.70375

**Published:** 2026-08-17

**Authors:** Juliana Schwaab, Paul Dolin, Bo Ding, Priya Jain, Lotte Westerink, Chris Edmonds, Tia Pennant, Oliver‐Thomas Carter, Fritha Hennessy, Stephanie Yanjing Chen

**Affiliations:** ^1^ Department of Hematology and Oncology, University Hospital Mannheim Heidelberg University Mannheim Germany; ^2^ BioPharmaceuticals Medical AstraZeneca Cambridge UK; ^3^ BioPharmaceuticals Medical AstraZeneca Gothenburg Sweden; ^4^ Health Economics & Payer Evidence AstraZeneca Cambridge UK; ^5^ Market Access and Pricing AstraZeneca Gaithersburg Maryland USA; ^6^ Adelphi Real World Bollington UK; ^7^ BioPharmaceuticals Medical AstraZeneca Gaithersburg Maryland USA

**Keywords:** biologic, disease burden, eosinophil, hypereosinophilic syndrome, idiopathic hypereosinophilic syndrome

## Abstract

**Introduction:**

Idiopathic hypereosinophilic syndrome (I‐HES) is a rare disorder characterized by persistent eosinophilia without an identifiable underlying cause, leading to organ damage and dysfunction. The objectives of this study were to describe the real‐world demographics, patient journey, treatment patterns, and disease burden of patients with I‐HES.

**Methods:**

Data were drawn from the Adelphi Real World HES Disease Specific Programme, a cross‐sectional survey of physicians and their patients with I‐HES in Europe (France, Germany, Italy, Spain, and the UK) and the United States from July to December 2023.

**Results:**

The overall population included 117 physicians and 451 patients. Patients were predominately male (62%), White (87%), and the mean (standard deviation [SD]) age was 44.7 (16.1) years. Mean (SD) and median (range) time between symptom onset and I‐HES diagnosis was 8.2 (11.1) and 4.2 (0–87.3) months, respectively. Most patients (66%) were treated with corticosteroids, and the mean (SD) and median (range) doses of oral and/or parenteral corticosteroids were 19.1 (20.3) and 10.0 (1.0–100.0) mg/day, respectively. The use of interleukin‐5/receptor alpha targeted therapies was low (23%). Patients had a mean (SD) of 7.7 (6.4) symptoms at diagnosis; 58% had organ system damage attributed to I‐HES, and disease was perceived by physicians as moderate or severe in 72%. Patient‐reported health‐related quality of life, work productivity, and fatigue were negatively impacted in patients with deteriorating or moderate‐to‐severe disease.

**Conclusion:**

These findings underscore the need for greater disease awareness to support timely diagnosis with use of targeted, corticosteroid‐sparing treatments to improve patient outcomes.

**Trial Registration:**

The authors have confirmed clinical trial registration is not needed for this submission.

## 1 Introduction

Hypereosinophilic syndrome (HES) is a group of rare disorders characterized by sustained hypereosinophilia in the blood and/or tissues leading to organ damage and dysfunction [1, 2, 3]. The clinical manifestations of HES are diverse: pulmonary, dermatologic, and gastrointestinal organ system involvement is most common in HES, though some patients experience neurologic and cardiac symptoms [4]. HES also varies in severity, ranging from an absence of symptoms to severe, life‐threatening complications [1, 2]. The diverse manifestations of HES can lead to a long patient journey, as patients experience multiple evaluations and referrals from healthcare professionals (HCPs) before receiving a diagnosis [5].

Based on pathophysiology and clinical presentation, HES is classified into primary or clonal (neoplastic), secondary (reactive), and idiopathic (I‐HES) subtypes [1]. Primary HES results from underlying stem cell, myeloid, or eosinophil neoplasms and is associated with genetic variations (e.g., in *PDGFRA/B*, *FGFR1*, or *JAK2*) [1, 6]. Secondary HES arises from a non‐neoplastic condition that causes cytokine‐driven hypereosinophilia and includes lymphocytic HES mediated by abnormal clonal T cells [1]. I‐HES, the most commonly diagnosed subtype [7], is characterized by sustained peripheral blood hypereosinophilia (≥ 1.5 × 10^9^/L on at least two occasions at least 4 weeks apart), with eosinophilic infiltration of tissues and organ dysfunction, without an identifiable underlying cause [1, 8]

I‐HES is generally treated with oral corticosteroids [6, 8]; however, long‐term corticosteroid usage is associated with an increased risk of developing complications including osteoporosis/osteoporotic fractures, cardiovascular disease, cerebrovascular disease, cataracts, type 2 diabetes, weight gain, renal impairment, infections such as pneumonia, and psychiatric disorders such as depression and anxiety [9, 10, 11]. Biologic therapies targeting interleukin‐5 (IL‐5) markedly reduce eosinophil counts by inhibiting eosinophil activation and differentiation [12] while those targeting the alpha subunit of the IL‐5 receptor (IL‐5Rα) induce eosinophil depletion via antibody‐dependent cell‐mediated cytotoxicity [13]. These therapies offer a pivotal advance in improving outcomes for patients with I‐HES. Currently, mepolizumab is the only anti‐IL‐5 therapy approved in the United States and Europe for the treatment of inadequately controlled I‐HES [14, 15]. Benralizumab, an anti‐IL‐5Rα therapy, was recently approved in the United States for the treatment of HES based on the efficacy and safety demonstrated in the phase 3 NATRON study (NCT04191304) [16, 17].

Data on the disease burden, health‐related quality of life (HRQoL), clinical management, and treatment patterns of patients with I‐HES are currently limited. The objectives of this study were to describe the real‐world demographics, patient journey, treatment patterns, and disease burden of patients with I‐HES across France, Germany, Italy, Spain, the United Kingdom, and the United States.

## 2 Methods

### 2.1 Study Design

This study forms part of the CONSTELLATION real‐world evidence program in rare, eosinophil‐associated diseases. Data were drawn from the Adelphi Real World HES Disease Specific Programme (DSP), a cross‐sectional survey (with retrospective data collection) of physicians and their patients with I‐HES in Europe (France, Germany, Italy, Spain, and the United Kingdom) and the United States from July to December 2023. DSPs are independent, impartial data sources and are not designed to address any pre‐specified hypotheses or research questions. The DSP methodology has been described previously [18, 19], validated [20], and demonstrated to be representative and consistent over time [21].

Participating physicians were first required to complete a physician survey focusing on their characteristics, patient caseload, and attitudes and perceptions regarding disease management and treatment alternatives. Each physician then completed patient record forms (PRFs) for 2–4 consecutively consulting patients with a diagnosis of I‐HES. PRFs were cross‐sectional; however, there was a retrospective element of data collection using physician‐reported information and details from patients’ medical records. The same patients for whom physicians completed a PRF were invited to independently complete a voluntary patient self‐completion survey (PSC).

PRFs and PSCs were linked via unique, pseudoanonymized physician and patient coded numbers. Additional information on the patient information collected from the surveys and physician recruitment can be found in the Supporting Information.

### 2.2 Study Population

Eligible physicians included rheumatologists, pulmonologists, allergists and immunologists, gastroenterologists, internal medicine specialists, dermatologists, otolaryngologists, cardiologists, neurologists, and hematologists who were responsible for the management and treatment of two or more patients with I‐HES.

Eligible patients were aged ≥ 10 years, with a physician‐confirmed diagnosis of I‐HES at the time of survey.

### 2.3 Statistical Methods

Descriptive statistics were used to summarize continuous variables (mean, standard deviation [SD], median, range, interquartile range [IQR]), and categorical variables (frequencies, percentages). As physicians could respond “don't know” in the PRF and questions in the PSC could be skipped, each variable was described using the maximum sample available to that variable. Missing data were not imputed.

## 3 Results

### 3.1 Physician Study Participation

A total of 117 physicians were enrolled in the survey, with hematologists being the most common specialty. Most physicians were managing ≥ 5 patients with I‐HES within the 12 months prior to survey date (Table 1).

**TABLE 1:** Physician study participation.

	Total physicians enrolled in the survey *N* = 117
**Physician specialty, *n* (%)**	
Hematologist	39 (33%)
Dermatologist	12 (10%)
Neurologist	12 (10%)
Pulmonologist	11 (9%)
Allergist/immunologist	10 (9%)
Gastroenterologist	10 (9%)
Internist	10 (9%)
Cardiologist	7 (6%)
Rheumatologist	5 (4%)
Otolaryngologist	1 (1%)
**Number of patients with I‐HES seen during the last 12 months,** ***n* (%)**	
< 5	32 (27%)
≥ 5	85 (73%)
**Total number of patients with I‐HES personally managed, *n* (%)**	
< 5	27 (23%)
≥ 5	90 (77%)

Abbreviation: I‐HES, idiopathic hypereosinophilic syndrome.

### 3.2 Patient Demographics

Data were reported by physicians in six countries on a total of 451 patients with I‐HES. Patient mean age was 44.7 years; over half were male and most were of White ethnicity (Table 2).

**TABLE 2:** Patient demographics and physician‐reported treatment for patients with I‐HES at survey date.

	Total patients with I‐HES *N* = 451
**Patient age (years)**	***n =* 449**
Mean (SD); [min, max]	44.7 (16.1); [11, 89]
**Patient sex, *n* (%)**	***n =* 451**
Male	278 (62%)
Female	173 (38%)
**Country, *n* (%)**	***n = 451***
France	84 (19%)
Germany	80 (18%)
Italy	87 (19%)
Spain	30 (7%)
UK	48 (11%)
US	122 (27%)
**Ethnicity, *n* (%)** ^a^, ^b^	***n =* 367**
White	319 (87%)
Black	24 (7%)
East or Southeast Asian	12 (3%)
Other^c^	13 (4%)
**PDGFRA, PDGFRB, FGFR1 molecular testing, *n* (%)**	***n* = 451**
Conducted at any point	195 (43%)
Conducted as part of I‐HES diagnosis	148 (33%)
**Treatment at survey date, *n* (%)** ^d^	***n =* 451**
**Corticosteroids**	**298 (66%)**
Oral corticosteroid	224 (50%)
Topical corticosteroid	73 (16%)
Inhaled/Nasal corticosteroid	44 (10%)
Parenteral corticosteroid	19 (4%)
**Immunosuppressants**	**52 (12%)**
Azathioprine	25 (6%)
Methotrexate	16 (4%)
Mycophenolate mofetil	8 (2%)
Cyclophosphamide	6 (1%)
**Biologics**	**172 (38%)**
Mepolizumab^e^	82 (18%)
Rituximab	32 (7%)
Dupilumab	25 (6%)
Benralizumab	16 (4%)
Omalizumab	10 (2%)
Reslizumab	6 (1%)
Tezepelumab	1 (<1%)
Hydroxyurea	28 (6%)
Tyrosine kinase inhibitor, such as imatinib	24 (5%)
Interferon	12 (3%)
IVIg	4 (1%)
PLEX	1 (<1%)
Other treatment	9 (2%)
None (current treatment)	17 (4%)
**Oral and/or parenteral corticosteroid dose (mg/day), *n***	***n =* 242**
Mean (SD); median [min, max]	19.1 (20.3); 10.0 [1, 100]
**Oral and/or parenteral corticosteroid dose (mg/day) (grouped),** ***n* (%)**	***n =* 242**
≤ 4	17 (7%)
4.1–7.5	81 (33%)
7.6–10	41 (17%)
11–20	38 (16%)
21–30	23 (10%)
31–40	6 (2%)
> 40	36 (15%)
**Duration of current regimen in patients prescribed oral corticosteroids with or without other treatments (months), *n***	***n* = 224**
Mean (SD); [min, max]	21.4 (39.0); [0, 454.2]
**Duration of current regimen in patients prescribed oral corticosteroids only (months), *n***	***n* = 103**
Mean (SD); [min, max]	24.7 (50.9); [0, 454.2]
**Symptomatic improvement since starting current treatment, *n* (%)** ^f^	***n =* 434**
Yes, symptomatic severity has improved	367 (85%)
No improvement	30 (7%)
Too early to tell	24 (6%)
Patient's condition has worsened since starting treatment	13 (3%)
**Symptoms that have shown improvement, *n* (%)** ^b^, ^g^	***n =* 367**
Fatigue	154 (42%)
Skin itching	126 (34%)
Skin rash	125 (34%)
Dyspnea, shortness of breath	109 (30%)
Cough	94 (26%)
General weakness	85 (23%)
Malaise	81 (22%)
Wheeze	80 (22%)
Urticaria	74 (20%)

Abbreviations: FGFR1, fibroblast growth factor receptor 1; I‐HES, idiopathic hypereosinophilic syndrome; IVIg, intravenous immunoglobulin; PDGFR, platelet‐derived growth factor receptor; PLEX, plasma exchange; SD, standard deviation.

^a^ Not asked in France.

^b^ Physicians could select multiple responses.

^c^ Includes South Asian, Middle Eastern, North African, Native Hawaiian, Pacific Islander, South or Central American Native, and American Indian, Indigenous American, or Alaska Native.

^d^ Patients may have been on ≥ 1 type of treatment.

^e^ At the time of data capture, mepolizumab was the only biologic approved for HES.

^f^ Physicians could select a single response.

^g^ Data only shown for symptoms showing improvement in ≥20% of patients.

### 3.3 Patient Journey

**FIGURE 1:**
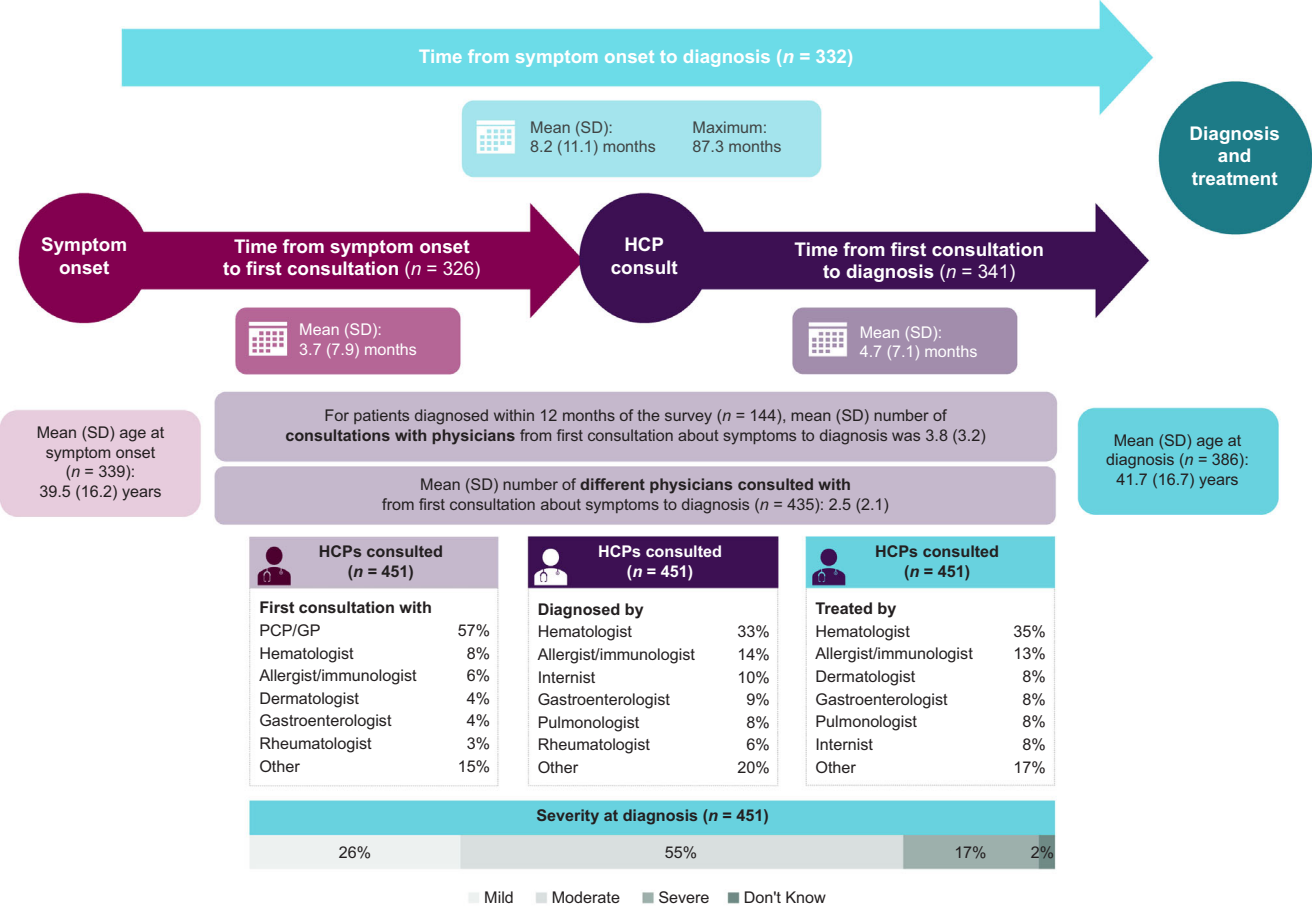
Patient journey from onset of I‐HES symptoms to diagnosis and treatment. No definitions of disease severity were provided in the survey, so these definitions were based on each physician's own judgement. GP, general practitioner; HCP, healthcare professional; I‐HES, idiopathic hypereosinophilic syndrome; PCP, primary care physician; SD, standard deviation.

Physicians reported delays between symptom onset and I‐HES diagnosis, including a mean of 3.7 months between symptom onset and first consultation with an HCP, and a mean of 4.7 months between first consultation and diagnosis. Overall, the mean time from symptom onset to diagnosis was 8.2 months (median 4.2 months), with some patients experiencing more substantial delays (up to 87.3 months). At diagnosis, physician‐perceived severity of I‐HES was moderate or severe in most patients (Figure 1).

Mean time from symptom onset to diagnosis varied across patient subgroups. Longer durations were observed among patients aged 56–65 years, those in the United Kingdom and Spain, those diagnosed by a gastroenterologist or primary care physician/general practitioner (PCP/GP), and those with a blood eosinophil count of < 300 cells/µL at diagnosis. Shorter durations were observed among patients aged < 35 years, those in Germany, those diagnosed by a rheumatologist, internist, neurologist, or cardiologist, and those with a blood eosinophil count ≥ 2500 cells/µL at diagnosis (Table S1).

### 3.4 Treatment Patterns

At survey completion, physicians reported two thirds of patients were receiving oral, parenteral, inhaled/nasal, or topical corticosteroids (prednisone, prednisolone, methylprednisolone, betamethasone, dexamethasone, or other). Of patients receiving oral and/or parenteral corticosteroids, the mean dose was 19.1 (median 10.0) mg/day (Table 2). Many patients were receiving high daily doses of oral and/or parenteral corticosteroids; 27% (*n =* 65/242) of patients were on > 20 mg/day (prednisone equivalent), of whom 55% (*n =* 36/65) were on > 40 mg/day. In patients receiving oral and/or parenteral corticosteroids with time since diagnosis data, the mean dose was greater in those diagnosed < 6 months prior to survey completion than those diagnosed ≥ 6 months prior to survey completion (Table S2). The mean duration of treatment in patients prescribed oral corticosteroids with or without other treatments was 21.4 months (Table 2).

Immunosuppressants were prescribed in 12% of patients and biologics in 38%. In total, 23% of patients were on an anti‐IL‐5/Rα treatment (primarily mepolizumab) (Table 2).

### 3.5 Disease Burden

#### 3.5.1 Physician‐Reported Disease Burden

**FIGURE 2:**
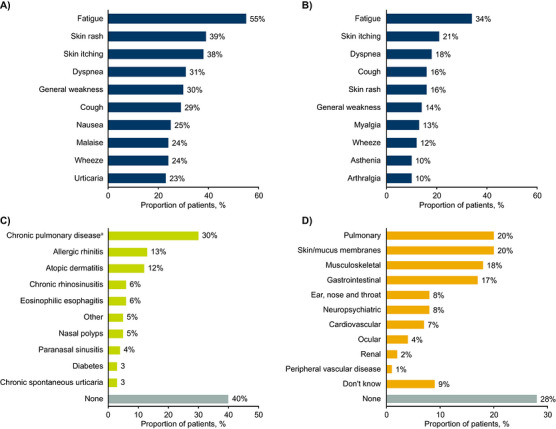
Physician‐reported 10 most common symptoms occurring in patients at I‐HES diagnosis (A) and survey completion (B), 10 most common concomitant conditions at survey completion (C), and organ system damage at survey completion (D) (*N* = 451). Physicians could select multiple responses. ^a^Chronic pulmonary disease included asthma, chronic obstructive pulmonary disease or other chronic pulmonary diseases. I‐HES, idiopathic hypereosinophilic syndrome.

Physician‐reported mean number of symptoms experienced by patients was 7.7 at diagnosis and 3.5 at survey completion (Table 3), and the average time from diagnosis at survey completion was 33.5 months. Fatigue was the most commonly reported symptom at diagnosis and survey completion, with skin itching also commonly reported at both time points (Figure 2). A full list of physician‐reported symptoms experienced by patients at diagnosis and survey completion is reported in Table S3.

**TABLE 3:** Physician‐reported disease burden of patients with I‐HES.

	Total patients with I‐HES *N =* 451
**Additional support/Care required, *n* (%)**	***n* = 451**
Yes	80 (18%)
No	347 (77%)
Don't know	24 (5%)
**Caregivers involved, *n* (%)** ^a^	***n =* 80**
Their partner/spouse	57 (71%)
Their parent/guardian	14 (18%)
Their child aged ≥18 years	10 (13%)
Their child aged <18 years	3 (4%)
Professional caregiver(s)	2 (3%)
Other non‐professional caregiver(s)	2 (3%)
Friend(s)/neighbor(s)	1 (1%)
Other relative(s)	1 (1%)
**Total number of symptoms experienced, mean (SD); [min, max]**	***n =* 451**
At diagnosis	7.7 (6.4); [0, 43]
At survey date	3.5 (3.9); [0, 26]
**Physician‐perceived severity of I‐HES at diagnosis, *n* (%)** ^b^, ^c^	***n =* 451**
Mild	118 (26%)
Moderate	247 (55%)
Severe	77 (17%)
Don't know	9 (2%)
**Physician‐perceived severity of I‐HES at survey date, *n* (%)** ^b^, ^c^	***n =* 451**
No active disease	122 (27%)
Mild	231 (51%)
Moderate	86 (19%)
Severe	12 (3%)
**Physician‐perceived expected progression of patients’ I‐HES, *n* (%)** ^b^, ^c^	***n =* 451**
Deteriorate rapidly	3 (1%)
Deteriorate moderately	8 (2%)
Deteriorate slowly	38 (8%)
Remain stable	160 (35%)
Improve slowly	115 (25%)
Improve moderately	65 (14%)
Improve rapidly	41 (9%)
Too early to determine	21 (5%)
**Peak blood eosinophil count (most recent), *n* (%)** ^b^	***n =* 262**
< 300 cells/µL	53 (20%)
300–499 cells/µL	90 (34%)
500–999 cells/µL	72 (27%)
1000–1499 cells/µL	30 (11%)
1500–2499 cells/µL	12 (5%)
≥2500 cells/µL	4 (2%)
Don't know	1 (<1%)

Abbreviations: I‐HES, idiopathic hypereosinophilic syndrome; SD, standard deviation.

^a^ Physicians could select multiple responses.

^b^ Physicians could select a single response.

^c^ No definitions of disease severity or disease progression were provided in the survey, so these definitions were based on each physician's own judgment.

The most common physician‐reported patient concomitant conditions were chronic pulmonary disease (including asthma, chronic obstructive pulmonary disease or other chronic pulmonary diseases), allergic rhinitis, and atopic dermatitis; 40% of patients had no reported concomitant conditions (Figure 2). A full list of physician‐reported patient concomitant conditions is reported in Table S4.

Overall, 58% (*n =* 263/451) of patients had organ system damage since their diagnosis of I‐HES. The most commonly reported organ system damage involved the pulmonary, skin/mucus membrane, and musculoskeletal systems. Just over a quarter of patients had not suffered any organ system damage (Figure 2).

While just over half of patients were considered by their physicians to have mild I‐HES at survey completion and more than a quarter were perceived to have no active disease. A total of 19% of patients were classified as having moderate disease and 3% as having severe disease (Table 3). The majority of patients with moderate disease and all patients with severe disease exhibited organ system damage. Even among patients perceived to have no active disease, 48% had organ system damage (Table S5).

In terms of physician‐perceived expectations of I‐HES progression, 35% of patients were expected to remain stable, 49% to improve, 11% to deteriorate, and for 5% of patients it was too early to predict (Table 3).

#### 3.5.2 Patient‐Reported Disease Burden

Overall, 27% of patients (*n =* 124/451) completed PSCs. The mean (SD) age of these patients was 38.5 (14.1) years, and the distribution of females (35%, *n =* 43/124) and males (65%, *n =* 81/124) was similar to that reported for the overall patient population.

A total of 41% of patients experienced I‐HES flares in the 12 months prior to survey completion, with a mean of 1.9 I‐HES flares among patients with a known number of flares.

Medication use was common among the 121 patients who provided responses on this, with only 7% not being prescribed any medication. In terms of treatment satisfaction, 84% of patients were satisfied or very satisfied with their current treatment (*n =* 90/107). While effective symptom control was the main reason for satisfaction, 44% highlighted that their medication enabled them to take part in social and family activities (Table S6).

**FIGURE 3:**
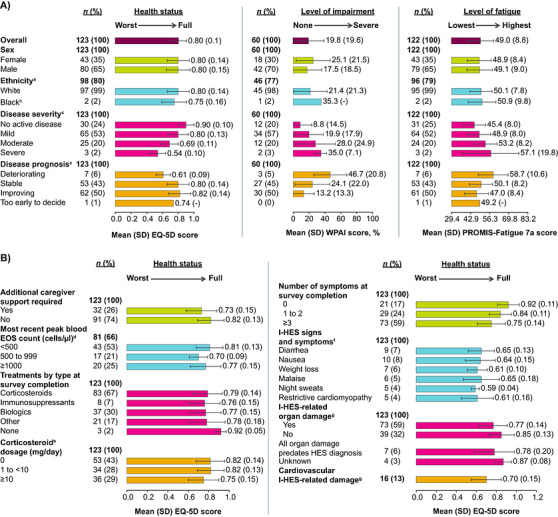
(A) Mean EQ‐5D, WPAI, and PROMIS‐Fatigue 7a scores, stratified by patient demographic characteristics and physician‐perceived I‐HES status and severity, and (B) mean EQ‐5D scores stratified by patient caregiver status, clinical and disease characteristics, and treatments at survey completion. ^a^Physicians were able to select more than one response for patient ethnicity. ^b^African American, African, or Caribbean. ^c^Definitions for disease severity and prognosis were based on physician judgement. ^d^One patient had an unknown most recent peak blood EOS count. Time since most recent test for blood EOS from data collection varied between patients: 73% <3 months, 17% ≥3–<6 months, 6% ≥6–<12 months, 4% ≥12 months. Ongoing treatment may have affected peak blood EOS count. ^e^Prednisolone equivalent. ^f^Signs and symptoms of I‐HES with an EQ‐5D score of ≤0.65 that impacted ≥5 patients who answered the EQ‐5D questionnaire. ^g^Organ damage was captured through medical records available to the physicians. EOS, eosinophil; EQ‐5D, EuroQol‐5 Dimension; I‐HES, idiopathic hypereosinophilic syndrome; PROMIS‐Fatigue 7a, Patient‐Reported Outcomes Measurement Information System Fatigue Short Form 7a; SD, standard deviation; WPAI, work productivity and activity impairment.

The EQ‐5D questionnaire provides a measure of HRQoL, with scores ranging from 0 (representing death) to 1 (indicating full health); higher scores reflect better quality of life [22, 23]. Among the 123 patients who completed the EQ‐5D, the mean (SD) age was 38.7 (14.0) years, with a similar sex distribution to the overall population (females: 35%, *n =* 43/123; males: 65%, *n =* 80/123). Mean EQ‐5D utility score (UK tariff) for patients was 0.80. EQ‐5D scores varied according to disease activity and clinical characteristics. The highest mean score was observed in patients with no active disease, whereas lower scores were observed among patients with moderate, severe, or deteriorating disease (0.69, 0.54, and 0.61, respectively). Lower scores were also observed among patients with cardiovascular I‐HES‐related damage, and patients who experienced symptoms such as diarrhea, nausea, weight loss, malaise, night sweats, and restrictive cardiomyopathy (Figure 3).

The WPAI questionnaire is used to measure the impact of health on work productivity and daily activities, with scores ranging from 0% (no impairment) to 100% (severe impairment) [24]. Among the 60 patients who completed the WPAI, the mean (SD) age was 39.1 (10.3) years, and there were more males (70%, *n =* 42/60) than females (30%, *n =* 18/60). Mean overall work impairment score was 19.8%. Patients with moderate, severe, or deteriorating disease had higher mean WPAI scores, while those with no active disease had a lower mean score (Figure 3).

The PROMIS‐Fatigue 7a questionnaire is used to assess the impact of fatigue on daily activities, with scores ranging from 29.4 (low level of fatigue) to 83.2 (high level of fatigue) [25]. A total of 122 patients completed the PROMIS‐Fatigue 7a questionnaire, with a mean (SD) age of 38.7 (14.2), and a similar sex distribution to the overall patient population (females: 35%, *n =* 43/122; males: 65%, *n =* 79/122). Mean PROMIS‐Fatigue score was 49.0. Patients with moderate, severe, or deteriorating disease had higher mean scores, and those with no active disease had a lower mean score (Figure 3).

#### 3.5.3 Physician Versus Patient Perceptions on Disease Burden

**FIGURE 4:**
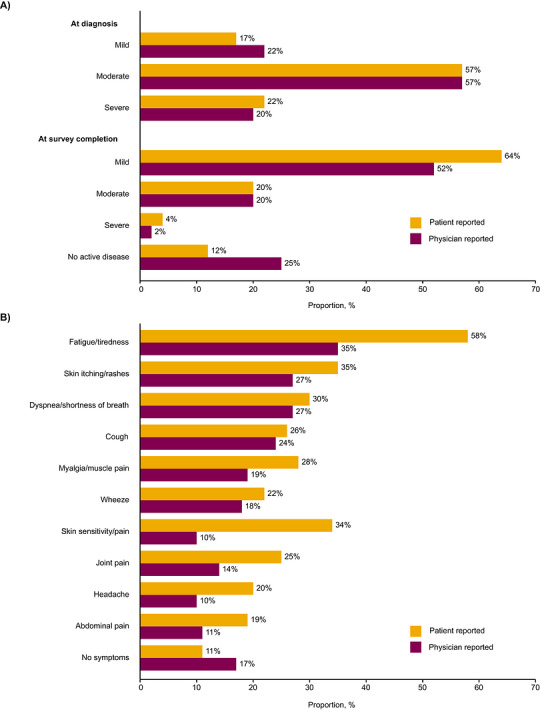
Matched physician‐ and patient‐reported data on (A) I‐HES severity at diagnosis and survey completion and (B) symptoms present at survey completion^a^. Physicians provided data for *n* = 124 patients for each question. Number of patients answering each question: *n* = 121 severity at diagnosis; *n* = 104 severity at survey completion; *n* = 122 symptoms experienced in the last 4 weeks. Definitions for disease severity were based on each physician's and patient's judgment. ^a^Physicians responded to the following question: “Which of the following symptoms is this patient currently experiencing?” Patients responded to the following question: “Which of the following symptoms have you experienced in the last 4 weeks?” I‐HES, idiopathic hypereosinophilic syndrome.

Physician‐provided patient data were matched to the equivalent patient‐provided data for 124 patients. Reporting of disease severity at diagnosis was largely similar between physicians and matched patients; however, a higher proportion of physicians reported that their patients had no active disease at survey completion compared with patients themselves (25% and 12%, respectively, Figure 4). To note, disease severity was based on subjective perception, as no objective or defined parameters were provided to physicians or patients.

The list of symptoms present at survey completion that physicians and patients could choose from differed, but there was considerable overlap. Fatigue was the most frequently reported symptom (58% of patients; 35% of physicians). Patients reported symptoms more frequently than physicians, including skin itching, dyspnea/shortness of breath, cough, myalgia/muscle pain, wheeze, skin sensitivity/pain, joint pain, headache, and abdominal pain. The proportion of physicians and patients who reported that patients had no symptoms was 17% and 11%, respectively (Figure 4).

### 3.6 Healthcare Resource Utilization

Physicians reported that 18% of patients visited the emergency room (ER) and 9% were hospitalized in the 12 months prior to survey completion. This resulted in an overall mean of 0.3 visits to the ER and 0.1 hospitalizations. Of the patients who had been hospitalized, the main reason was to treat a complication of I‐HES. Most patients who had been hospitalized were perceived to have moderate or severe I‐HES at diagnosis. Pulmonary damage was the most common organ system damage in those hospitalized, followed by gastrointestinal and skin/mucus membrane damage (Table S7).

Of patients with ≥ 1 hospitalization stratified by physician‐perceived disease severity, 64% had severe disease, 17% had moderate disease, 6% had mild disease, and 4% had no active disease (Table S8).

## 4 Discussion

In this study, we provide real‐world data on the patient journey, treatment patterns, and disease burden for patients with I‐HES in Europe and the United States.

The mean time from the onset of clinical symptoms to I‐HES diagnosis was 8 months, less than the 22–24 months reported previously [5, 26]. This shorter time to diagnosis may be due to factors such as the physicians included in this study being more familiar with the condition, as they were specialists who were managing multiple patients with I‐HES. Given that the average time from diagnosis at survey completion was 33.5 months, recall bias may have impacted the time to diagnosis reported. Early symptoms of I‐HES are non‐specific (e.g., fatigue) and may have initially been associated with other conditions [1]. Patients were also considerably symptomatic by the time of diagnosis, with most having physician‐perceived moderate or severe disease, which could have shortened time to diagnosis. However, even with a shorter mean time to diagnosis, the maximum time from symptom onset to diagnosis in our study was high (i.e., 87.3 months), and patients needed to consult multiple different physicians before being diagnosed with I‐HES.

In this study, patients who received treatment were mostly prescribed corticosteroids, consistent with previous studies [4, 7]. In patients treated with oral and/or parenteral corticosteroids, the majority received high daily doses, with almost 30% receiving doses > 20 mg/day, which far exceeds recommended doses [8]. In addition, the average duration of oral corticosteroid use (with or without other treatments) was prolonged, at approximately 21 months. As expected, daily corticosteroid doses were highest for patients diagnosed < 6 months prior to survey completion as they were in a dose‐tapering phase; nonetheless, doses were considerably high among patients expected to have completed tapering, which may have been due to patients increasing their doses to achieve symptomatic relief. Even though corticosteroids are the mainstay of I‐HES treatment [2, 6, 8], high doses and cumulative exposure are associated with a higher risk of adverse effects including osteoporosis/osteoporotic fractures, cardiovascular disease, cerebrovascular disease, and others [9, 10, 11, 27, 28], highlighting the ongoing need for alternative treatments. In our study, less than a quarter of patients received anti‐IL‐5/Rα treatment, despite mepolizumab being approved for treatment of HES at the time of study conduct [14, 15, 29, 30].

We found substantial disease burden and organ system damage among patients with I‐HES. Over 80% of patients experienced symptoms at survey completion and 58% suffered organ system damage, reflecting the typical clinical manifestation of I‐HES [31]. Furthermore, nearly half the patients perceived by their physicians as having inactive disease also exhibited organ damage, indicating that long‐term consequences of I‐HES may persist despite the absence of active symptoms.

EQ‐5D data from this study demonstrate a decline in quality of life with increasing I‐HES severity and the substantial impact of increasing disease activity on daily functioning and well‐being. In terms of other patient‐reported outcomes, many patients expressed satisfaction with their treatment because it enabled participation in social and family activities, which are domains central to well‐being and daily life.

When matching PRF and PSC responses, reporting of disease severity was largely similar between physicians and patients; however, more physicians reported their patients had no active disease at survey completion compared with the patients themselves. These discrepancies could be due to treatments suppressing eosinophil counts (which physicians may interpret as no active disease) but not fully resolving symptoms. Fatigue, a hallmark symptom of I‐HES, was still experienced by most patients in our study, despite treatment. This was underestimated by the physicians, highlighting that fatigue needs more careful assessment as it is not usually a symptom that is clinically observable.

The symptoms reported in our study population are comparable to those recently published by Khoury et al. [32] in which the authors developed and assessed a patient reported outcome measure to better capture symptom experiences of patients with HES. Both studies support the value of patient‐reported outcome measures for capturing patients’ subjective experiences in both clinical studies and daily routine.

While ER visits and hospitalizations in our study appear to be low, it is important to contextualize these results: these data were from a short period (12 months prior to survey completion), but we saw an increasing trend in the proportion of patients hospitalized with increasing perceived disease severity, suggesting the disease may have a considerable healthcare resource utilization burden.

A key strength of this study is that it provides real‐world data on a large cohort of patients with I‐HES, adding to the limited evidence base surrounding I‐HES. However, this study was subjected to limitations. The study population was not a true random sample, and although minimal selection criteria were imposed to identify physicians for inclusion, participation was influenced by the willingness to complete the record forms. Misdiagnosis may have been possible as I‐HES is a rare disease, and differential diagnosis of I‐HES through immunophenotyping and/or molecular/genetic testing was not a requirement (nor were results of these tests collected for patients who were tested). In addition, no patient selection verification procedures were applied to the HES DSP, and identification of the patients included was based on physician judgment/perception and not independently verified. Moreover, patients included were actively seeking medical consultation and therefore may have been more severely affected by their disease. As PSC completion was voluntary, the possibility of non‐response bias cannot be excluded, and patient‐reported symptoms, HRQoL, fatigue, and work productivity outcomes should be interpreted in light of this. The retrospective nature of information capture may introduce recall bias; however, physicians could refer to patients’ medical records while completing the PRF, minimizing this possibility. As there are no validated tools to measure disease activity in I‐HES and no definitions of mild, moderate, or severe disease, respondents were left to define these categories subjectively. It is also not known by which criteria the physicians and patients considered these terms (e.g., if hospitalizations were part of a respondent's definition of severe disease), so these caveats should be kept in mind when interpreting data on disease severity. Some subgroups (e.g., patients diagnosed by cardiologists) had a small sample size, which could skew interpretation of results.

## 5 Conclusion

I‐HES is a complex disorder with heterogenous clinical presentation, and this real‐world analysis highlights the substantial burden associated with I‐HES, including diagnostic delays, high corticosteroid dependence and frequent organ involvement, even among patients classified as having inactive disease. Despite current treatment approaches, many patients continue to experience persistent symptoms and impaired HRQoL, underscoring that treatment benefits must extend beyond clinical control to meaningful improvements in quality of life, and that HRQoL measures should be incorporated into clinical decision making and future research. The discordance observed between physician‐ and patient‐reported symptoms further highlights the importance of incorporating the patient perspective into disease assessment and monitoring. There is a need for improved management strategies that reduce reliance on corticosteroids while supporting better management of disease activity, symptom burden, and long‐term complications. Clinical studies will help to further evaluate the role of early intervention with targeted biologic agents in such management strategies, given their established efficacy in reducing eosinophils and improving clinical outcomes. These findings also reinforce the importance of continuous disease monitoring for all patients with I‐HES, as a purely symptom‐based or episodic approach may overlook ongoing pathology that adversely affects long‐term outcomes. These results also underscore the importance of increasing disease awareness among healthcare providers to support earlier diagnosis and intervention, with the goal of reducing irreversible organ damage and improving patient outcomes. Enhanced awareness may also contribute to optimizing healthcare resource utilization and mitigating the substantial costs associated with managing advanced disease manifestations and complications.

## Author Contributions

All authors contributed equally to this work and gave their consent for the publication of this article.

## Funding

This study was funded by AstraZeneca.

## Ethics Statement

Data collection was undertaken in line with European Pharmaceutical Marketing Research Association guidelines as all data were aggregated and de‐identified before receipt. As such, the study did not require ethics committee approval. Each survey was performed in full accordance with relevant legislation at the time of data collection, including the US Health Insurance Portability and Accountability Act 1996 (https://www.hhs.gov/hipaa/for‐professionals/privacy/laws‐regulations/index.html), the European Pharmaceutical Marketing Research Association guidelines (https://www.ephmra.org/sites/default/files/2024‐09/2024), and Health Information Technology for Economic and Clinical Health Act legislation (https://www.healthit.gov/sites/default/files/hitech_act_excerpt_from_arra_with_index.pdf).

## Consent

The authors have nothing to report.

## Conflicts of Interest

The authors declare the following financial interests/relationships which may be considered as a potential conflict of interest:

Juliana Schwaab declares receiving consultancy fees from AstraZeneca, GSK, Novartis and Blueprint, research funding from GSK and Blueprint, and honoraria from GSK, Blueprint, AstraZeneca, Novartis, Abbvie, and AOP. Paul Dolin, Bo Ding, Priya Jain, Lotte Westerink, Chris Edmonds, and Stephanie Yanjing Chen are or were employees or contractors of AstraZeneca at the time of study conduct and may own stock/stock options. Tia Pennant, Oliver‐Thomas Carter, and Fritha Hennessy are or were employees of Adelphi Real World, which received funding from AstraZeneca to conduct this analysis.

## Supporting information




**Supporting Information**: jha270375‐sup‐0001‐SuppMat.docx

## Data Availability

The methodology, materials, data, and data analysis that support the findings in this manuscript are the intellectual property of Adelphi Real World and were used under license and are thus not publicly available. All requests for data should be addressed directly to Fritha Hennessy of Adelphi Real World at fritha.hennessy@omc.com.
