# Supplementary material for: Patient Journey, Treatment Patterns, and Disease Burden of Patients With Idiopathic Hypereosinophilic Syndrome
Source: EJHaem. 2026 Aug 17;7(4):e70375. doi: 10.1002/jha2.70375 (PMC13481312; doi:10.1002/jha2.70375)
Supplement: Supplementary file 1 [file JHA2-7-e70375-s001.docx]

# Supplementary Materials

[Contents	1](#_Toc223430270)

[Supplementary Methods	2](#_Toc223430271)

[Supplementary Tables	3](#_Toc223430272)

[TABLE S1 | Physician-reported duration between onset of I-HES symptoms and diagnosis by various subgroup	3](#_Toc223430273)

[TABLE S2 | Physician-reported oral and/or parenteral corticosteroid dose stratified by time since diagnosis	5](#_Toc223430274)

[TABLE S3 | Physician-reported symptoms experienced by patients with I-HES at diagnosis and survey completion	6](#_Toc223430275)

[TABLE S4 | Full list of physician-reported patient concomitant conditions	8](#_Toc223430276)

[TABLE S5 | Organ system damage stratified by physician-perceived severity of I-HES	10](#_Toc223430277)

[TABLE S6 | Patient-reported outcomes	11](#_Toc223430278)

[TABLE S7 | Healthcare resource utilization	16](#_Toc223430279)

[TABLE S8. Number of hospitalizations and HCP visits stratified by physician-perceived I-HES severity at survey completion	19](#_Toc223430280)

[References	21](#_Toc223430281)

## Supplementary Methods

**Patient Information Collected**

The physician-completed patient record form (PRF) collected information including: demographics; time to I-HES diagnosis; physician involvement in diagnosis and treatment; I-HES treatment; caregiver involvement; symptoms; organ system damage; concomitant conditions; physicians’ impressions of disease severity and expected progression (as a formal definition of I-HES disease severity is lacking); and healthcare resource utilization.

Information collected in the patient self-completion survey (PSC) included: caregiver support (where applicable); symptoms and severity; treatment, effect of treatment on symptoms, and treatment satisfaction; and I-HES flares experienced in the last 12 months. Additionally, three patient-reported outcome measures were captured: EuroQol-5 Dimension (EQ-5D) UK tariff [1-3]; Work Productivity and Activity Impairment (WPAI) [4]; and Patient-Reported Outcomes Measurement Information System^®^ Fatigue Short Form 7a (PROMIS-Fatigue 7a) [5].

**Physician Recruitment**

A representative sample of physicians were recruited to participate in the Adelphi Real World HES Disease Specific Programme^TM^ by local fieldwork agents, following completion of a short screening questionnaire. Physician participation was financially incentivized, with reimbursement upon survey completion according to fair market research rates.

## Supplementary Tables

### TABLE S1 | Physician-reported duration between onset of I-HES symptoms and diagnosis by various subgroup

|  | **Patients with  I-HES in subgroup  (*n =* 332), *n* (%)** | **Months from symptom onset to diagnosis, mean (SD)** |
| --- | --- | --- |
| **Biological sex** | | |
| Male | 211 (64%) | 8.3 (11.5%) |
| Female | 121 (36%) | 8.1 (10.5%) |
| **Patient age, years** | | |
| 0–17 | 7 (2%) | 3.4 (3.1%) |
| 18–25 | 40 (12%) | 5.3 (4.5%) |
| 26–35 | 64 (19%) | 6.5 (6.0%) |
| 36–45 | 89 (27%) | 9.6 (12.3%) |
| 46–55 | 55 (17%) | 7.9 (12.1%) |
| 56–65 | 45 (14%) | 11.6 (16.0%) |
| 66–75 | 19 (6%) | 8.1 (11.1%) |
| ≥76 | 13 (4%) | 8.9 (11.7%) |
| **Region** | | |
| France | 71 (21%) | 7.6 (9.2%) |
| Germany | 66 (20%) | 4.2 (3.4%) |
| Italy | 62 (19%) | 9.8 (12.8%) |
| Spain | 25 (8%) | 11.9 (11.8%) |
| UK | 36 (11%) | 13.5 (19.8%) |
| US | 72 (22%) | 7.3 (7.8%) |
| **Diagnosing physician** | | |
| Hematologist | 117 (35%) | 7.2 (9.6%) |
| Rheumatologist | 25 (8%) | 5.8 (7.2%) |
| Pulmonologist | 26 (8%) | 8.5 (10.1%) |
| Internist | 42 (13%) | 6.9 (5.9%) |
| Allergist/immunologist | 35 (11%) | 8.1 (9.6%) |
| Gastroenterologist | 29 (9%) | 16.4 (21.1%) |
| PCP/GP | 16 (5%) | 10.7 (15.7%) |
| Dermatologist | 22 (7%) | 9.4 (10.9%) |
| Neurologist | 17 (5%) | 5.2 (4.6%) |
| Cardiologist | 3 (1%) | 1.7 (1.5%) |
| **Blood eosinophil count at diagnosis (cells/µL)** | | |
| <300 cells/µL | 7 (2%) | 31.9 (33.2%) |
| 300–499 cells/µL | 11 (3%) | 10.5 (17.0%) |
| 500–999 cells/µL | 27 (8%) | 8.5 (9.0%) |
| 1000–1499 cells/µL | 52 (16%) | 9.7 (9.1%) |
| 1500–2499 cells/µL | 133 (40%) | 7.2 (9.7%) |
| ≥2500 cells/µL | 95 (29%) | 7.0 (9.1%) |
| Collected, but no record of blood  eosinophil count | 4 (1%) | 3.7 (3.8%) |
| Not collected | 3 (1%) | 5.8 (6.6%) |

Abbreviations: GP, general practitioner; I-HES, idiopathic hypereosinophilic syndrome; PCP, primary care physician; SD, standard deviation.

### TABLE S2 | Physician-reported oral and/or parenteral corticosteroid dose stratified by time since diagnosis

|  | **Time since diagnosis** | | |
| --- | --- | --- | --- |
|  | **<6 months prior  to survey** | **≥6 months prior  to survey** | **Total** |
| **Oral and/or parenteral corticosteroid dose (mg/day)** | ***n =* 43** | ***n =* 168** | ***n =* 211** |
| Mean (SD); [min, max] | 33.3 (24.6); [1, 90] | 16.0 (17.6), [1, 80] | 19.5 (20.4); [1, 90] |
| **Oral and/or parenteral corticosteroid dose (mg/day) (grouped), *n* (%)** | ***n =* 43** | ***n =* 168** | ***n =* 211** |
| ≤4 | 3 (7%) | 13 (8%) | 16 (8%) |
| 4.1–7.5 | 4 (9%) | 64 (38%) | 68 (32%) |
| 7.6–10 | 5 (12%) | 33 (20%) | 38 (18%) |
| 11–20 | 8 (19%) | 20 (12%) | 28 (13%) |
| 21–30 | 3 (7%) | 19 (11%) | 22 (10%) |
| 31–40 | 4 (9%) | 2 (1%) | 6 (3%) |
| >40 | 16 (37%) | 17 (10%) | 33 (16%) |

Abbreviation: SD, standard deviation.

### TABLE S3 | Physician-reported symptoms experienced by patients with I-HES at diagnosis and survey completion

| **Symptoms experienced  by patients, *n* (%)** | **At  diagnosis (*n =* 451)** | **At survey completion  (*n =* 451)** | **Symptoms experienced  by patients, *n* (%)** | **At  diagnosis (*n =* 451)** | **At survey completion  (*n =* 451)** |
| --- | --- | --- | --- | --- | --- |
| None | 18 (4%) | 87 (19%) | Tingling, burning, or numbness in extremities | 40 (9%) | 23 (5%) |
| Fatigue | 247 (55%) | 154 (34%) | Redness, irritation | 39 (9%) | 16 (4%) |
| Skin rash | 176 (39%) | 74 (16%) | Joint stiffness | 38 (8%) | 18 (4%) |
| Skin itching | 170 (38%) | 95 (21%) | Trouble with balance, coordination | 30 (7%) | 21 (5%) |
| Dyspnea, shortness of breath | 139 (31%) | 79 (18%) | Arthritis, joint swelling | 28 (6%) | 6 (1%) |
| General weakness | 136 (30%) | 61 (14%) | Decreased range of motion | 27 (6%) | 9 (2%) |
| Cough | 131 (29%) | 74 (16%) | Skin hardening, induration | 25 (6%) | 9 (2%) |
| Nausea | 111 (25%) | 34 (8%) | Hair loss | 23 (5%) | 6 (1%) |
| Malaise | 110 (24%) | 28 (6%) | Cognitive difficulties | 22 (5%) | 16 (4%) |
| Wheeze | 108 (24%) | 55 (12%) | Hard to gain weight | 20 (4%) | 9 (2%) |
| Urticaria | 104 (23%) | 38 (8%) | Loss of elasticity | 18 (4%) | 10 (2%) |
| Asthenia, lack of energy | 101 (22%) | 47 (10%) | Joint redness | 18 (4%) | 3 (1%) |
| Myalgia, muscle pain | 94 (21%) | 60 (13%) | Weakness in one or more extremities | 18 (4%) | 11 (2%) |
| Weight loss | 90 (20%) | 30 (7%) | Constipation | 17 (4%) | 11 (2%) |
| Abdominal pain | 88 (20%) | 40 (9%) | Ear pain, drainage | 15 (3%) | 12 (3%) |
| Fever | 86 (19%) | 13 (3%) | Myocarditis (acute) | 14 (3%) | 2 (<1%) |
| Diarrhea | 83 (18%) | 35 (8%) | Diarrhea – bloody | 13 (3%) | 10 (2%) |
| Arthralgia, joint pain | 75 (17%) | 44 (10%) | Cardiomyopathy | 13 (3%) | 5 (1%) |
| Night sweats | 68 (15%) | 20 (4%) | Carpal tunnel syndrome | 12 (3%) | 4 (1%) |
| Skin inflammation | 67 (15%) | 19 (4%) | Skin grooves or farrows along paths of veins | 11 (2%) | 3 (1%) |
| Runny nose | 62 (14%) | 24 (5%) | Puckered skin, orange peel texture | 9 (2%) | 5 (1%) |
| Heart palpitations | 61 (14%) | 34 (8%) | Weight gain | 9 (2%) | 5 (1%) |
| Upper abdominal discomfort (bloating, tightness, fullness) | 60 (13%) | 18 (4%) | Otitis media | 9 (2%) | 1 (<1%) |
| Vomiting | 60 (13%) | 9 (2%) | Ascites | 8 (2%) | 1 (<1%) |
| Headache | 60 (13%) | 35 (8%) | Blurry vision | 8 (2%) | 6 (1%) |
| Loss of appetite | 59 (13%) | 22 (5%) | Refusal to eat | 7 (2%) | – |
| Difficulty swallowing | 54 (12%) | 27 (6%) | Restrictive cardiomyopathy | 6 (1%) | 6 (1%) |
| Skin pain | 54 (12%) | 22 (5%) | Scleroderma (localized) | 5 (1%) | 5 (1%) |
| Chills | 51 (11%) | 7 (2%) | Other | 4 (1%) | 5 (1%) |
| Sinusitis | 49 (11%) | 21 (5%) | Endocarditis | 4 (1%) | 2 (<1%) |
| Skin thickening | 46 (10%) | 20 (4%) | Endocardial fibrosis | 3 (1%) | 3 (1%) |
| Skin swelling (angioedema) | 45 (10%) | 10 (2%) | Poor growth (infants, children) | 3 (1%) | 2 (<1%) |
| Sinus congestion (pain, pressure) | 43 (10%) | 26 (6%) | Myocarditis fibrosis | 3 (1%) | 3 (1%) |
| Chest pain | 42 (9%) | 18 (4%) | Scleroderma (systemic) | 2 (<1%) | 1 (<1%) |
| Dizziness, light-headedness | 40 (9%) | 20 (4%) | Endocarditis (acute) | 1 (<1%) | – |

Abbreviation: I-HES, idiopathic hypereosinophilic syndrome.

### TABLE S4 | Full list of physician-reported patient concomitant conditions

|  | **Total patients with I-HES  *N =* 451** |
| --- | --- |
| **Concomitant conditions, *n* (%)^a^** | ***n =* 451** |
| No concomitant conditions^b^ | 180 (40%) |
| Chronic pulmonary disease^c^ | 134 (30%) |
| Allergic rhinitis | 57 (13%) |
| Atopic dermatitis | 52 (12%) |
| Chronic rhinosinusitis | 27 (6%) |
| Eosinophilic esophagitis | 26 (6%) |
| Other | 21 (5%) |
| Nasal polyps | 21 (5%) |
| Paranasal sinusitis | 17 (4%) |
| Diabetes without chronic complications | 15 (3%) |
| Chronic spontaneous urticaria | 15 (3%) |
| Congestive heart failure | 13 (3%) |
| Renal disease | 12 (3%) |
| Hand and/or foot eczema | 12 (3%) |
| Peripheral vascular disease | 12 (3%) |
| Peptic ulcer disease | 11 (2%) |
| Long term effects of COVID-19 | 11 (2%) |
| Mild liver disease | 10 (2%) |
| Myocarditis | 10 (2%) |
| Polyneuropathy | 10 (2%) |
| Pulmonary infiltrates | 10 (2%) |
| Other eosinophilic gastrointestinal disease | 9 (2%) |
| Psoriasis | 8 (2%) |
| Vasculitis | 7 (2%) |
| Myocardial infarction | 7 (2%) |
| Ulcerative colitis | 6 (1%) |
| Multiple mononeuropathy | 5 (1%) |
| Rheumatologic disease | 5 (1%) |
| Inflammatory bowel disease | 5 (1%) |
| Cerebrovascular disease | 5 (1%) |
| Diabetes with chronic complications | 5 (1%) |
| Psoriatic arthritis | 4 (1%) |
| AIDS/HIV | 3 (1%) |
| Dementia | 2 (<1%) |
| Crohn’s disease | 2 (<1%) |
| Any malignancy, including lymphoma | 1 (<1%) |
| Moderate or severe liver disease | 1 (<1%) |

Abbreviations: AIDS, acquired immunodeficiency syndrome; HIV, human immunodeficiency virus; I-HES, idiopathic hypereosinophilic syndrome.

^a^Physicians could select multiple responses.

^b^Patients who were reported to have neither inflammatory nor non-inflammatory concomitant conditions.

^c^Includes patients diagnosed with asthma, chronic obstructive pulmonary disease, or other chronic pulmonary diseases.

### TABLE S5 | Organ system damage stratified by physician-perceived severity of I-HES

|  | **Physician-perceived severity^a^ (at survey date)** | | | |
| --- | --- | --- | --- | --- |
|  | **No active disease *n =* 122 (27%)** | **Mild *n =* 231 (51%)** | **Moderate *n =* 86 (19%)** | **Severe *n =* 12 (3%)** |
| **Organ system damage, n (%)^b^** | | | | |
| Overall | 59 (48%) | 178 (77%) | 75 (87%) | 12 (100%) |
| Pulmonary | 10 (8%) | 47 (20%) | 25 (29%) | 7 (58%) |
| Skin/mucus membranes | 19 (16%) | 51 (22%) | 12 (14%) | 7 (58%) |
| Musculoskeletal | 14 (11%) | 43 (19%) | 23 (27%) | 2 (17%) |
| Gastrointestinal | 12 (10%) | 42 (18%) | 22 (26%) | 2 (17%) |
| Don’t know | 15 (12%) | 20 (9%) | 7 (8%) | – |
| Ear/nose/throat | 8 (7%) | 18 (8%) | 11 (13%) | 1 (8%) |
| Neuropsychiatric | – | 19 (8%) | 16 (19%) | – |
| Cardiovascular | 4 (3%) | 15 (6%) | 10 (12%) | 2 (17%) |
| Ocular | 4 (3%) | 8 (3%) | 6 (7%) | 2 (17%) |
| Renal | – | 6 (3%) | 1 (1%) | 1 (8%) |
| Peripheral vascular disease | – | 3 (1%) | – | 1 (8%) |
| Other | – | – | 2 (2%) | – |
| This patient does not have any organ damage | 63 (52%) | 53 (23%) | 11 (13%) | – |
| All organ damage this patient has predates their diagnosis | 2 (2%) | 12 (5%) | 4 (5%) | 1 (8%) |

Abbreviation: I-HES, idiopathic hypereosinophilic syndrome.

^a^Definitions for disease severity were based on physician judgement.

^b^Physicians could select multiple responses.

### TABLE S6 | Patient-reported outcomes

| **Variable** | **Total patients with I-HES who completed PSCs *n =* 124** |
| --- | --- |
| **Time between symptom onset and first consultation (days)** | ***n =* 113** |
| Mean (SD); [min, max] | 126.0 (218.7); [0, 1460] |
| **Age at symptom onset (years)** | ***n =* 123** |
| Mean (SD); [min, max] | 32.9 (14.0); [1, 73] |
| **Symptom severity at diagnosis, *n* (%)^a^** | ***n =* 121** |
| Mild | 21 (17%) |
| Moderate | 69 (57%) |
| Severe | 27 (22%) |
| Don't know | 4 (3%) |
| **Symptom severity at survey date, *n* (%)^a^** | ***n =* 121** |
| Not present | 24 (21%) |
| Mild | 68 (58%) |
| Moderate | 21 (18%) |
| Severe | 4 (3%) |
| **I-HES worsened since diagnosis, *n* (%)** | ***n =* 122** |
| Yes | 9 (7%) |
| No | 108 (89%) |
| Don't know | 5 (4%) |
| **I-HES symptoms at survey date vs 12 months ago, *n* (%)^a^** | ***n =* 123** |
| Much worse | 1 (1%) |
| Slightly worse | 5 (4%) |
| Neither worse nor better | 24 (20%) |
| Slightly better | 44 (36%) |
| Much better | 49 (40%) |
| **Symptoms at survey date, *n* (%)**^b^ | ***n =* 122** |
| Fatigue/tiredness | 71 (58%) |
| Skin rashes/itchiness | 43 (35%) |
| Skin sensitivity/pain | 42 (34%) |
| Shortness of breath | 36 (30%) |
| Muscle pain | 34 (28%) |
| Persistent cough | 32 (26%) |
| Joint pain | 31 (25%) |
| Wheezing | 27 (22%) |
| Skin discoloration | 25 (20%) |
| Headache | 25 (20%) |
| Abdominal pain | 23 (19%) |
| Diarrhea | 18 (15%) |
| Trouble breathing (general) | 17 (14%) |
| Chest pain | 17 (14%) |
| Loss of appetite | 16 (13%) |
| Feeling bloated | 14 (11%) |
| Numbness in hands and/or feet | 14 (11%) |
| Difficulty sleeping | 14 (11%) |
| Fast heartbeat | 13 (11%) |
| Irritation/redness in eye(s) | 13 (11%) |
| Skin thickening/hardening | 13 (11%) |
| Loss of strength in hands and/or feet | 12 (10%) |
| Increased discharge from nose | 12 (10%) |
| Blocked sinuses | 11 (9%) |
| Joint stiffness | 11 (9%) |
| Weight loss | 10 (8%) |
| Heartburn | 10 (8%) |
| Burning sensation in hands and/or feet | 10 (8%) |
| Trouble breathing (through nose) | 10 (8%) |
| Anxiety | 9 (7%) |
| Pins and needles in hands and/or feet | 9 (7%) |
| Feeling sick/vomiting | 8 (7%) |
| Ear fullness/blockage | 8 (7%) |
| Dizziness | 7 (6%) |
| High blood pressure | 7 (6%) |
| Loss of sense of smell | 6 (5%) |
| Swelling of eye(s) | 6 (5) |
| Pain in ears | 6 (5%) |
| Heart pain | 6 (5%) |
| Fever | 5 (4%) |
| Chills/sweats | 4 (3%) |
| Skin ulcer/hives | 4 (3%) |
| Hair loss | 4 (3%) |
| Forgetfulness/confusion | 3 (2%) |
| Presence of blood in stools | 3 (2%) |
| Blurred vision | 3 (2%) |
| Weight gain | 2 (2%) |
| Pain in eye | 2 (2%) |
| Facial pain | 2 (2%) |
| Difficulties with coordination | 2 (2%) |
| Redness in urine/darker urine/red urine | 1 (1%) |
| Other symptom(s) | 1 (1%) |
| No symptoms | 13 (11%) |
| **I-HES flares in the last 12 months, *n* (%)** | ***n =* 124** |
| Yes | 51 (41%) |
| No | 69 (56%) |
| Don't know | 4 (3%) |
| **Number of I-HES flares in the last 12 months^c^** | ***n =* 49** |
| Mean (SD); [min, max] | 1.9 (1.1); [1, 6] |
| **Caregiver support required, *n* (%)** | ***n =* 122** |
| Yes | 15 (12%) |
| No | 107 (88%) |
| **Work/study changed due to I-HES, *n* (%)^b^** | ***n =* 124** |
| I have not changed how I work/study or I don't work/study | 106 (85%) |
| I had to change my job/study arrangements | 8 (6%) |
| I had to reduce the number of hours I work/study | 6 (5%) |
| I had to retire from working/stop studying | 2 (2%) |
| I'm on long term sick leave | 2 (2%) |
| **Number of individual medications prescribed, *n* (%)^a^** | ***n =* 121** |
| 0 | 8 (7%) |
| 1 | 72 (60%) |
| 2 | 30 (25%) |
| ≥3 | 11 (9%) |
| **Effect of medication on symptoms, *n* (%)^a^** | ***n =* 113** |
| Significantly worse | 1 (1%) |
| Slightly worse | – |
| Neither better nor worse | 12 (11%) |
| Slightly better | 36 (32%) |
| Significantly better | 64 (57%) |
| **Satisfaction with current medication, *n* (%)^a^** | ***n =* 107** |
| Very dissatisfied | 1 (1%) |
| Dissatisfied | – |
| Neither satisfied nor dissatisfied | 16 (15%) |
| Satisfied | 52 (49%) |
| Very satisfied | 38 (36%) |
| **Reasons for satisfaction with current medication, *n* (%)^b^** | ***n =* 90** |
| The medication reduces my symptoms in the way that I would like it to | 68 (76%) |
| The medication allows me to engage in social and family activities | 40 (44%) |
| The medication is easy to administer/take | 39 (43%) |
| I only take one/a small number of medications to help with my I-HES | 39 (43%) |
| The medication has little to no side effects | 27 (30%) |
| The medication does not cost that much | 23 (26%) |
| I feel the benefits of the medication shortly after taking it | 17 (19%) |
| I do not have to take the medication that frequently | 17 (19%) |
| The medication helps with my other conditions | 9 (10%) |
| Another reason | 1 (1%) |
| **Reasons for dissatisfaction with current medication, *n* (%)^b^** | ***n =* 1** |
| The medication does not reduce my symptoms in the way I would like it to | 1 (100%) |
| I am still unable to engage fully in social and family activities | 1 (100%) |
| It takes a while for me to feel the benefits of the medication | 1 (100%) |

Abbreviations: I-HES, idiopathic hypereosinophilic syndrome; PSC, patient self-completion survey; SD, standard deviation.

^a^Physicians could select a single response.

^b^Physicians could select multiple responses.

^c^Patients with a known number of I-HES flares.

### TABLE S7 | Healthcare resource utilization

|  | **Total patients with I-HES  *N =* 451** |
| --- | --- |
| **Number of ER visits due to I-HES in the last 12 months, *n* (%)** | ***n =* 368** |
| Mean (SD); [min, max] | 0.3 (0.8); [0, 6] |
| No ER visits | 302 (82%) |
| 1 ER visit due to I-HES | 44 (12%) |
| 2 ER visits due to I-HES | 13 (4%) |
| 3 ER visits due to I-HES | 3 (1%) |
| 4 ER visits due to I-HES | 2 (<1%) |
| 5 ER visits due to I-HES | 2 (<1%) |
| 6 ER visits due to I-HES | 2 (<1%) |
| **Number of hospitalizations due to I-HES in the last 12 months, *n* (%)** | ***n =* 373** |
| Mean (SD); [min, max] | 0.1 (0.5); [0, 5] |
| None | 339 (91%) |
| 1 | 29 (8%) |
| 2 | 2 (<1%) |
| 3 or more | 3 (<1%) |
| **Number of times hospitalization involved treating a I-HES flare** | ***n =* 34** |
| Mean (SD); [min, max] | 0.9 (0.6); [0, 3] |
| **Reason for most recent hospitalization due to I-HES, *n* (%)**^a^ | ***n =* 34** |
| To treat a complication of I-HES | 29 (85%) |
| To manage an adverse treatment reaction | 3 (9%) |
| Other | 1 (3%) |
| Don’t know | 1 (3%) |
| **Number of times hospitalization involved treating a new manifestation** | ***n =* 34** |
| Mean (SD); [min, max] | 0.2 (0.5); [0, 2] |
| **Number of times hospitalization involved treating another complication** | ***n =* 34** |
| Mean (SD); [min, max] | 0.3 (0.4); [0, 1] |
| **Time spent in hospital, *n* (%)** | ***n =* 34** |
| Nights | 24 (71%) |
| Day case | 7 (21%) |
| Don't know | 3 (9%) |
| **Nights spent in hospital** | ***n =* 24** |
| Mean (SD); [min, max] | 4.8 (3.5); [1, 16] |
| **HCP-perceived severity of I-HES at diagnosis in patients who were hospitalized, *n* (%)^a,b^** | ***n =* 34** |
| Mild | 3 (9%) |
| Moderate | 15 (44%) |
| Severe | 15 (44%) |
| Don’t know | 1 (3%) |
| **HCP-perceived severity of I-HES at survey completion in patients who were hospitalized, *n* (%)^a,b^** | ***n =* 34** |
| No active disease | 4 (12%) |
| Mild | 11 (32%) |
| Moderate | 12 (35%) |
| Severe | 7 (21%) |
| **Organ system damage in patients who were hospitalized, *n* (%)^c^** | ***n =* 34** |
| Pulmonary | 13 (38%) |
| Gastrointestinal | 10 (29%) |
| Skin/mucus membranes | 8 (24%) |
| Musculoskeletal | 7 (21%) |
| Ears/nose/throat | 5 (15%) |
| Neuropsychiatric | 4 (12%) |
| Cardiovascular | 4 (12%) |
| Ocular | 3 (9%) |
| Peripheral vascular disease | 2 (6%) |
| All organ damage this patient has predates their I-HES diagnosis | 3 (9%) |
| This patient does not have any organ damage | 3 (9%) |
| Don’t know | 2 (6%) |

Abbreviations: ER, emergency room; HCP, healthcare professional; I-HES, idiopathic hypereosinophilic syndrome; SD, standard deviation.

^a^Physicians could select a single response.

^b^Definitions for disease severity were based on physician judgement.

^c^Physicians could select multiple responses.

### TABLE S8. Number of hospitalizations and HCP visits stratified by physician-perceived I-HES severity at survey completion

|  | **Physician-perceived severity^a^ (at survey date)** | | | | **Total** |
| --- | --- | --- | --- | --- | --- |
|  | **No active disease** | **Mild** | **Moderate** | **Severe** |  |
| **Number of hospitalizations, *n* (%)** | ***n* = 104** | ***n* = 188** | ***n* = 70** | ***n* = 11** | ***n* = 373** |
| None, *n* (%) | 100 (96%) | 177 (94%) | 58 (83%) | 4 (36%) | 339 (91%) |
| ≥1, *n* (%) | 4 (4%) | 11 (6%) | 12 (17%) | 7 (64%) | 34 (9%) |
| Mean (SD); [min, max] | 0.04 (0.2); [0, 1] | 0.1 (0.3); [0, 3] | 0.2 (0.6); [0, 4] | 1.3 (1.7); [0, 5] | 0.1 (0.5); [0, 5] |
| **Visits to different HCPs** |  |  |  |  |  |
| **Hematologist, *n* (%)** | **53 (27%)** | **106 (55%)** | **28 (15%)** | **6 (3%)** | **193 (100%)** |
| Mean (SD); [min, max] | 7.1 (4.7); [2, 20] | 6.3 (4.3); [1, 30] | 5.0 (3.3); [1, 12] | 4.5 (2.7); [2, 9] | 6.3 (4.3); [1, 30] |
| **Pulmonologist, *n* (%)** | **16 (21%)** | **34 (44%)** | **24 (31%)** | **4 (5%)** | **78 (100%)** |
| Mean (SD); [min, max] | 3.8 (1.6); [2, 8] | 2.4 (1.1); [1, 4] | 2.9 (1.4); [1, 6] | 2.5 (1.0); [2, 4] | 2.8 (1.4); [1, 8] |
| **Allergist/Immunologist, *n* (%)** | **14 (19%)** | **40 (55%)** | **16 (22%)** | **3 (4%)** | **73 (100%)** |
| Mean (SD); [min, max] | 3.2 (1.5); [2, 5] | 2.5 (1.4); [1, 6] | 2.9 (1.6); [1, 6] | 4.7 (2.5); [2, 7] | 2.8 (1.6); [1, 7] |
| **Dermatologist, *n* (%)** | **21 (32%)** | **29 (44%)** | **15 (23%)** | **1 (2%)** | **66 (100%)** |
| Mean (SD); [min, max] | 3.3 (2.2); [1, 12] | 4.1 (3.3); [1, 13] | 2.9 (2.0); [1, 7] | 1.0 (0.0); [1, 1] | 3.5 (2.7); [1, 13] |
| **Gastroenterologist, *n* (%)** | **15 (23%)** | **37 (56%)** | **12 (18%)** | **2 (3%)** | **66 (100%)** |
| Mean (SD); [min, max] | 3.0 (1.3); [1, 6] | 4.1 (4.6); [1, 20] | 4.0 (2.8); [2, 10] | 1.5 (0.7); [1, 2] | 3.8 (3.7); [1, 20] |
| **Internist, *n* (%)** | **19 (30%)** | **26 (41%)** | **14 (22%)** | **4 (6%)** | **63 (100%)** |
| Mean (SD); [min, max] | 3.0 (1.2); [2, 6] | 3.0 (1.8); [1, 8] | 4.0 (1.6); [2, 8] | 2.8 (2.1); [1, 5] | 3.2 (1.6); [1, 8] |
| **GP/PCP, *n* (%)** | **14 (24%)** | **34 (58%)** | **10 (17%)** | **1 (2%)** | **59 (100%)** |
| Mean (SD); [min, max] | 4.0 (1.8), [2, 8] | 3.6 (3.0), [1, 16] | 4.2 (3.5), [1, 12] | 2.0 (0), [2, 2] | 3.8 (2.8); [1, 16] |
| **Neurologist, *n* (%)** | **5 (9%)** | **31 (56%)** | **18 (33%)** | **1 (2%)** | **55 (100%)** |
| Mean (SD); [min, max] | 2.8 (1.3), [1, 4] | 4.0 (2.5), [2, 12] | 3.7 (2.8), [1, 12] | 3.0 (0), [3, 3] | 3.8 (2.5); [1, 12] |
| **Cardiologist, *n* (%)** | **6 (11%)** | **31 (57%)** | **16 (30%)** | **1 (2%)** | **54 (100%)** |
| Mean (SD); [min, max] | 3.3 (1.2), [2, 5] | 2.2 (1.1), [1, 5] | 2.4 (1.7), [1, 6] | 4.0 (0), [4, 4] | 2.4 (1.4), [1, 6] |
| **Rheumatologist, *n* (%)** | **13 (31%)** | **20 (48%)** | **8 (19%)** | **1 (2%)** | **42 (100%)** |
| Mean (SD); [min, max] | 2.2 (1.1), [1, 4] | 3.4 (1.2), [1, 6] | 3.9 (2.2), [1, 8] | 3.0 (0), [3, 3] | 3.1 (1.5), [1, 8] |
| **Otolaryngologist, *n* (%)** | **2 (13%)** | **8 (53%)** | **5 (33%)** | **0** | **15 (100%)** |
| Mean (SD); [min, max] | 2.0 (0), [2, 2] | 3.3 (1.2), [2, 5] | 2.6 (1.1), [1, 4] | – | 2.9 (1.1), [1, 5] |
| **Nurse, *n* (%)** | **0** | **4 (36%)** | **5 (45%)** | **2 (18%)** | **11 (100%)** |
| Mean (SD); [min, max] | – | 2.8 (1.3), [1, 4] | 11.2 (5.4), [2, 16] | 3.0 (1.4), [2, 4] | 6.6 (5.6), [1, 16] |
| **Nephrologist, *n* (%)** | **1 (11%)** | **4 (44%)** | **3 (33%)** | **1 (11%)** | **9 (100%)** |
| Mean (SD); [min, max] | 2.0 (0), [2, 2] | 2.0 (1.4), [1, 4] | 1.3 (0.6), [1, 2] | 3.0 (0), [3, 3] | 1.9 (1.1), [1, 4] |
| **Emergency department physician, *n* (%)** | **0** | **0** | **4 (80%)** | **1 (20%)** | **5 (100%)** |
| Mean (SD); [min, max] | – | – | 2.0 (1.4), [1, 4] | 1.0 (0), [1, 1] | 1.8 (1.3), [1, 4] |

Abbreviations: GP, general practitioner; HCP, healthcare professional; I-HES, idiopathic hypereosinophilic syndrome; PCP, primary care physician; SD, standard deviation.

^a^Definitions for disease severity were based on physician judgement.

## References

1.	P. Dolan, "Modeling valuations for EuroQol health states," *Med Care* 35 (1997): 1095-1108, <https://doi.org/10.1097/00005650-199711000-00002>.

2.	M. Herdman, C. Gudex, A. Lloyd, et al., "Development and preliminary testing of the new five-level version of EQ-5D (EQ-5D-5L)," *Qual Life Res* 20 (2011): 1727-1736, <https://doi.org/10.1007/s11136-011-9903-x>.

3.	D. Rowen, C. Mukuria, N. Bray, et al., "UK valuation of EQ-5D-5L, a generic measure of health-related quality of life: a study protocol," *Value Health* 26 (2023): 1625-1635, <https://doi.org/10.1016/j.jval.2023.08.005>.

4.	M.C. Reilly, A.S. Zbrozek, E.M. Dukes, "The validity and reproducibility of a work productivity and activity impairment instrument," *Pharmacoeconomics* 4 (1993): 353-365, <https://doi.org/10.2165/00019053-199304050-00006>.

5.	PROMIS. PROMIS Fatigue scoring manual 2019. Date last accessed: June 30, 2025.
